# 

**Published:** 1888-06

**Authors:** 


RECEIPTED—1888—Drs. N. C Pyles, T J. Jones, J B. Bailey to July; P. R. Cortilyou, Dr. Gann to Oct; C. B. Thomas to April; John Thomas to April Robt. Norton, Wm. Sterne, James L Johnson, T. S. Lawton, R. M. Browne, R. L. Simpson, E. E. Meyers, T. L. Quillian, G. W. Wright, J. M. Boling, to May; B. M. Wooley, to January.

FOR 1887—F. C. Davis, C. M. Curtis, Jas. Oliver, N, J. Simonton, J. T, Ballenger, C. J. Ducote, to June.

FOR 1889—L. C. Goneks to April.

SANDERS & SONS' EUCALYPTI EXTRACT (*Eucalyptol*).—Apply to Dr. Sanders, Dillon, Iowa, for gratis supplied reports on cures effected at the clinics of the Universities of Bonn and Griefswald.

